# Antihypertensive Effects of *Lindera erythrocarpa* Makino via NO/cGMP Pathway and Ca^2+^ and K^+^ Channels

**DOI:** 10.3390/nu16173003

**Published:** 2024-09-05

**Authors:** Sujin Shin, Junkyu Park, Ho-Young Choi, Youngmin Bu, Kyungjin Lee

**Affiliations:** 1 Department of Korean Medicine, Graduate School, Kyung Hee University, Seoul 02447, Republic of Korea; sjshin04@khu.ac.kr; 2 Department of Science in Korean Medicine, Graduate School, Kyung Hee University, Seoul 02447, Republic of Korea; ojeoksan@khu.ac.kr; 3 Department of Herbal Pharmacology, College of Korean Medicine, Kyung Hee University, Seoul 02447, Republic of Korea; hychoi@khu.ac.kr (H.-Y.C.); ymbu@khu.ac.kr (Y.B.)

**Keywords:** *Lindera erythrocarpa*, blood pressure, hypertension, hypotensive effect, spontaneously hypertensive rat, vasorelaxant, endothelium, NO/cGMP pathway, angiotensin II

## Abstract

Studies have demonstrated the therapeutic effects of *Lindera* plants. This study was undertaken to reveal the antihypertensive properties of *Lindera erythrocarpa* leaf ethanolic extract (LEL). Aorta segments of Sprague–Dawley rats were used to study the vasodilatory effect of LEL, and the mechanisms involved were evaluated by treating specific inhibitors or activators that affect the contractility of blood vessels. Our results revealed that LEL promotes a vasorelaxant effect through the nitric oxide/cyclic guanosine 3′,5′-monophosphate pathway, blocking the Ca^2+^ channels, opening the K^+^ channels, and inhibiting the vasoconstrictive action of angiotensin II. In addition, the effects of LEL on blood pressure were investigated in spontaneously hypertensive rats by the tail-cuff method. LEL (300 or 1000 mg/kg) was orally administered to the rats, and 1000 mg/kg of LEL significantly lowered the blood pressure. Systolic blood pressure decreased by −20.06 ± 4.87%, and diastolic blood pressure also lowered by −30.58 ± 5.92% at 4 h in the 1000 mg/kg LEL group. Overall, our results suggest that LEL may be useful to treat hypertensive diseases, considering its vasorelaxing and hypotensive effects.

## 1. Introduction

Cardiovascular diseases (CVDs) are major noncommunicable diseases (NCDs) and account for approximately 50% of the NCD-related deaths [1]. Among the risk factors for CVDs, hypertension is one of the most compelling indicators of a causal relationship and is associated with a higher frequency of exposure [2]. Maintaining normal blood pressure is crucial; however, hypertension is often deemed an inevitable part of aging, with a prevalence of 60–75% in older adults (≥60 years) [3,4]. With the rapidly aging population, the prevalence of hypertension is anticipated to escalate further [5]. However, a recent study found that more than half of the patients with hypertension in the United States have uncontrolled blood pressure [6]. Therefore, new therapeutic and preventive strategies are needed to manage patients with high blood pressure.

Medicinal plants have emerged as potential agents for managing CVDs and have been proven to be beneficial in reducing cardiovascular risk factors [7]. The therapeutic potential of plants is mainly attributed to their bioactive compounds, including polyphenols, dietary fibers, and carotenoids [8,9]. In particular, plants and their natural compounds have shown hypotensive effects by promoting vasorelaxation through various mechanisms, which include stimulating the nitric oxide (NO)/cyclic guanosine 3′,5′-monophosphate (cGMP) signaling pathway, inhibiting angiotensin-converting enzymes, or blocking the Ca^2+^ channels [10,11]. Additionally, several commercially available drugs for CVDs, including ephedrine, digitoxin, and salicin, originate from plants [12]. Although many plants and their natural compounds have demonstrated benefits in managing hypertension, their specific mechanisms of action remain unknown. Therefore, further research is required to gain a deeper understanding of their inherent complexities.

The genus *Lindera* holds economic significance due to its utilization in teas, spices, and fragrances. Furthermore, *Lindera* exhibits notable medicinal and therapeutic effects attributed to its diverse pharmacological properties [13,14]. Studies have demonstrated the anti-hypertensive effects of *Lindera* species [15,16]. *L. erythrocarpa* (LE) has been used as an herbal remedy for treating digestive disorders, alleviating thirst, and managing pain [17]. Recent studies have revealed various components of LE, such as linderone, lucidone, kanakuziol, camphene, and limonene, showcasing their diverse effects including anti-inflammatory, anticancer, and antifungal activities [18]. However, no studies have investigated the anti-hypertensive effects of LE. Therefore, this study investigated the vascular effects of LE leaf 50% ethanol extract (LEL) on aorta rings of Sprague–Dawley (SD) rats under various experimental conditions. In addition, the hypotensive effect of LEL oral administration in spontaneously hypertensive rats (SHRs) was assessed.

## 2. Materials and Methods

### 2.1. Preparation of the Extract

LEL samples were collected from Seogwipo-si, Jeju, Republic of Korea, in June 2023 and authenticated by Prof. Ho-Young Choi in the Department of Herbal Pharmacology, College of Korean Medicine, Kyung Hee University. The LEL samples were deposited at the College of Korean Medicine, Kyung Hee University, Seoul, Republic of Korea. Dried and grinded LEL samples were extracted by boiling them with 50% ethanol for 2 h at 70 ± 2 °C. After filtration, the extract was freeze-dried. The extract yield of LEL was 18.57% (extract yield = weight of the final freeze-dried extract/weight of the dried sample × 100).

### 2.2. Animals

Male SD rats (6–7 weeks old) were provided by Daehan Bio Link (Eumseong-gun, Republic of Korea), and male SHRs (16–17 weeks old) were provided by SLC, Inc. (Shizuoka, Japan). Animals were housed under standard laboratory conditions with free access to food and tap water. All experimental protocols were approved by the Institutional Animal Care and Use Committee of Kyung Hee University (approval number: KHSASP-23-506 on 17 November 2023).

### 2.3. Chemicals and Solution Preparation

Dimethyl sulfoxide (DMSO) was obtained from Junsei (Tokyo, Japan). Phenylephrine (PE), acetylcholine (ACh), indomethacin, methylene blue (MB), ethyleneglycol-bis(2-aminoethylether)-N,N‚N,’N′-tetraacetic acid (EGTA), Bay K8644, and angiotensin II were provided by Sigma Aldrich (St. Louis, MA, USA). N^G^-nitro-L-arginine methyl ester (L-NAME), 4-aminopyridine (4-AP), and tetraethylammonium (TEA) were purchased from Wako Pure Chemical Industries (Osaka, Japan). 1H-[1,2,4] oxadiazolo [4,3-a]quinoxalin-1-one (ODQ) was purchased from the Tokyo Chemical Industry (Tokyo, Japan). All other reagents were provided by Daejeong Chemical & Gold (Siheung-si, Republic of Korea). 

The composition of the Krebs Henseleit (KH) solution, mM, was as follows: NaCl, 118; KCl, 4.7; KH_2_PO_4_, 1.2; MgSO_4_, 1.2; CaCl_2_, 2.5; NaHCO_3_, 25; and glucose, 11.1.

### 2.4. Ex Vivo Vasorelaxant Evaluation

#### 2.4.1. General Experimental Procedures

The vasodilatory effect was evaluated following the established methods [19]. Briefly, the SD rats were sacrificed with urethane (1.2 g/kg, intraperitoneal injection). The thoracic aorta was extracted and was cleaned of the adjacent and connective tissue. Multiple aortic rings from one SD rat were cut to the same length and assigned to either the control or experimental group to minimize intergroup differences. Each ring was considered an experimental unit. Rings were mounted between hooks with a tension of 1.0 g in glass chambers containing KH solution bubbled with an O_2_/CO_2_ (95:5%) mixture and maintained at 37 °C. According to reliable statistics and animal ethics principles, we set the sample size to at least four per group, as described previously [20].

#### 2.4.2. Role of Endothelium in LEL-Induced Vasorelaxant Activity

The endothelium-intact rings were assessed by relaxation (>85%) to ACh (10 μM) in the rings constricted with PE (1 μM). To process the endothelium-removed rings, the intimal surface of the artery was mechanically removed by rubbing with a smooth wooden stick. The endothelium-removed rings were assessed by relaxation (<10%) to ACh (10 μM). After confirming the endothelium, the rings were induced contraction by PE (1 μM). Following the attainment of the maximum blood vessel constriction by PE, the cumulative concentrations of LEL dissolved in DMSO were applied to KH buffer in the glass chamber containing endothelium-intact or endothelium-removed aorta. 

#### 2.4.3. Role of NO Synthase and Cyclooxygenase (COX) in LEL-Induced Vasorelaxant Activity

The thoracic aortic rings were incubated with L-NAME (an NO synthase inhibitor, 100 μM) or indomethacin (a COX inhibitor, 10 μM) for 20 min. After incubation, constriction of the rings was induced by PE (1 μM). Following the attainment of the maximum blood vessel contraction by PE, the cumulative concentrations of LEL were applied (0.5, 1, 2, 5, and 10 μg/mL).

#### 2.4.4. Role of Soluble Guanylate Cyclase (sGC) and cGMP in LEL-Induced Vasorelaxant Activity

ODQ (an sGC inhibitor, 10 μM) or MB (a cGMP inhibitor, 10 μM) were applied to the aortic rings and incubated for 20 min. After incubation, contraction of the rings was induced by PE (1 μM). Following the attainment of the maximum blood vessel constriction by PE, the cumulative concentrations of LEL were applied (0.5, 1, 2, 5, and 10 μg/mL).

#### 2.4.5. Effect of LEL on Ca^2+^ Influx through Ca^2+^ Channels

The aortic rings were stabilized in a Ca^2+^-free KH solution with EGTA (1 mM). They were treated with LEL (100, 200, 500, and 1000 μg/mL) for 20 min and, subsequently, PE (1 μM) for 20 min. After incubation, the cumulative concentrations of CaCl_2_ were applied.

#### 2.4.6. Effect of LEL on Rings Constricted by Bay K8644

Rings were treated with LEL (1000 μg/mL) for 20 min. After incubation, Bay K8644 solution (L-type Ca^2+^ channel agonist, 10 µM) was introduced in each chamber.

#### 2.4.7. Role of K^+^ Channels in LEL-Induced Vasorelaxant Activity

BaCl_2_ (inward rectifier K^+^ channel blocker, 10 μM), 4-AP (voltage-dependent K^+^ channel blocker, 1 mM) or TEA (large-conductance Ca^2+^-activated K^+^ channel blocker, 1 mM) were applied to the aortic rings and left to incubate for 20 min. Then, contraction of the rings was induced by PE (1 μM). Following the attainment of the maximum blood vessel contraction by PE, the cumulative concentrations of LEL were applied (0.5, 1, 2, 5, and 10 μg/mL).

#### 2.4.8. Effect of LEL on Rings Constricted by Angiotensin II

The rings were treated with LEL (10 μg/mL) for 20 min. After incubation, angiotensin II (10^−9^−10^−6^ M) was applied to each chamber.

### 2.5. Blood Pressure Measurement

SHRs were acclimated by measuring the blood pressure once a day for 1 week before the experiment. SHRs with systolic blood pressure (SBP) < 190 mmHg and diastolic blood pressure (DBP) < 140 mmHg were excluded from the study. Eighteen SHRs were randomly allocated into three groups for oral administration as follows: the control group (orally administered distilled water) and LEL groups at two different levels (orally administered 300 mg/kg and 1000 mg/kg of body weight dissolved in distilled water). Blood pressure was measured as previously described [19]. To minimize potential confounders, the blood pressure of one or two rats per group was measured simultaneously. The control group rats whose blood pressure was not maintained (difference of >20% from that measured before administration) were excluded from the analysis.

### 2.6. Statistical Analysis

Statistical analyses were performed using multiple unpaired *t*-tests and two-way analysis of variance with multiple comparisons (simple effects within rows), followed by Dunnett’s post hoc test. Differences between the experimental and control groups were evaluated at the same concentrations or times. GraphPad Prism software version 9 (San Diego, CA, USA) was used for statistical procedures. In all the analyses, *p* < 0.05 was considered significant.

## 3. Results

### 3.1. Role of Endothelium in LEL-Induced Vasorelaxant Activity

**Figure 1:**
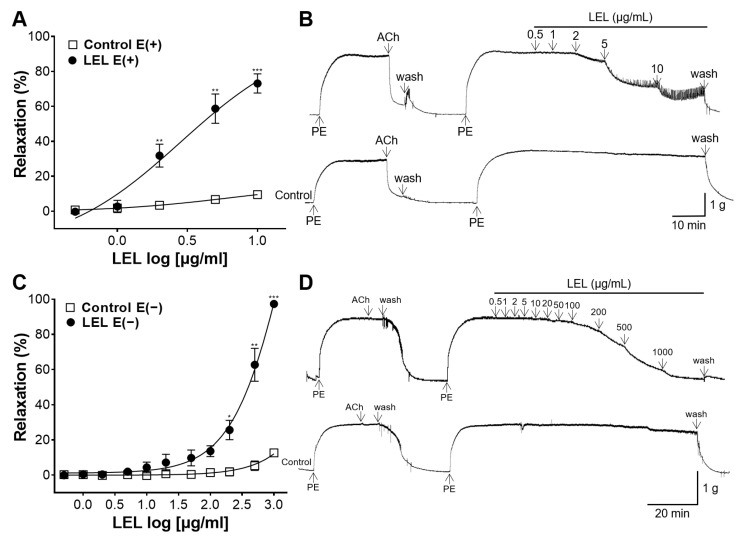
Effect of *Lindera erythrocarpa* leaf 50% ethanol extract (LEL) on endothelium-intact [E(+)] or endothelium-absent [E(−)] aorta, which underwent induced constriction with phenylephrine (PE, 1 μM). To confirm the endothelium, relaxation of pre-constricted aortas was induced by acetylcholine (ACh, 10 μM). (**A**,**C**) Relaxation response to LEL and (**B**,**D**) representative tracing. Data shown are the mean ± standard error of the mean (SEM) (*n* = 5). * *p* < 0.05, ** *p* < 0.01, *** *p* < 0.001 vs. control.

LEL significantly relaxed the endothelium-intact aorta compared with the control in a dose-dependent manner, and the E_max_ value was 73.07 ± 5.50% at 10 μg/mL (Figure 1A,B). LEL also significantly relaxed the endothelium-absent rings, and the E_max_ value was 97.29 ± 0.39% at 1000 μg/mL (Figure 1C,D).

### 3.2. Role of NO Synthase and COX in LEL-Induced Vasorelaxant Activity

**Figure 2:**
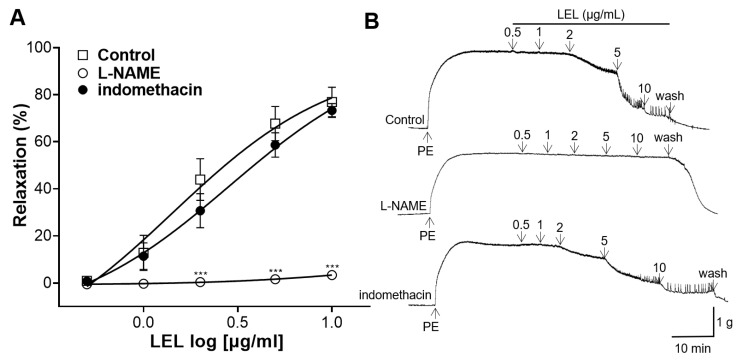
Effect of LEL on aortic rings which underwent induced constriction by PE (1 μM) without (Control) or with the incubation of L-NAME (100 μM) or indomethacin (10 μM). (**A**) Relaxation response to LEL and (**B**) representative tracing. Data shown are the mean ± SEM (*n* = 5–6). *** *p* < 0.001 vs. control.

To elucidate the involvement of NO synthase and COX in LEL-induced relaxation, the aortic rings were incubated with L-NAME, a NO synthase inhibitor, or indomethacin, a COX inhibitor. LEL treatment with the incubation of L-NAME resulted in the reduced relaxation of the rings contracted by PE compared to the control; however, incubation with indomethacin did not reveal any significant effects on LEL-induced vasorelaxation (Figure 2).

### 3.3. Role of sGC and cGMP in LEL-Induced Vasorelaxant Activity

**Figure 3:**
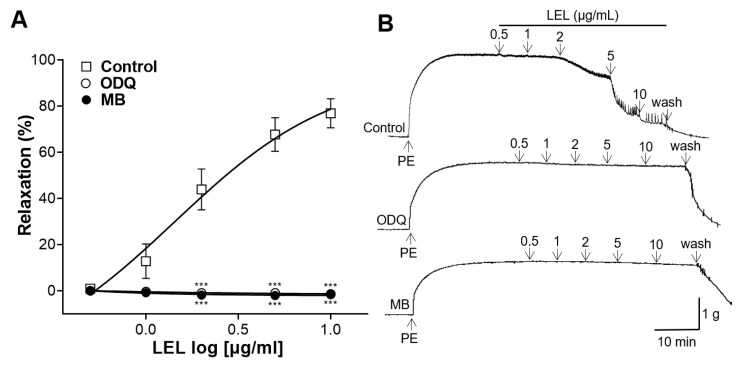
Effect of LEL on aortic rings with induced contraction by PE (1 μM), without (Control) or with the incubation of ODQ (10 μM) or MB (10 μM). (**A**) Relaxation response to LEL and (**B**) representative tracing. Data shown are the mean ± SEM (*n* = 5–6). *** *p* < 0.001 vs. control.

To determine the role of sGC and cGMP in LEL-induced vasorelaxant activity, the rings were incubated with ODQ, an sGC inhibitor, or MB, a cGMP inhibitor. LEL resulted in a significantly reduced relaxation of aortic rings in the presence of ODQ and MB (Figure 3).

### 3.4. Effect of LEL on Ca^2+^ Influx

**Figure 4:**
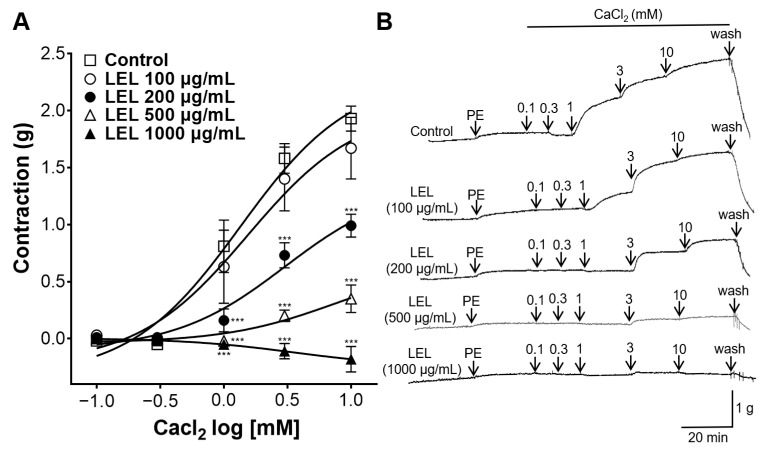
Effect of LEL on Ca^2+^ influx in the thoracic aorta. The rings were treated with PE (1 μM) before the constriction caused by CaCl_2_. (**A**) Contraction response to CaCl_2_ and (**B**) representative tracing. Data shown are the mean ± SEM (*n* = 4–6). *** *p* < 0.001 vs. control.

To elucidate the effect of LEL on Ca^2+^ influx via Ca^2+^ channels, the rings were constricted by CaCl_2_. LEL (200, 500, and 1000 μg/mL) significantly reduced the contraction of aortic rings. The maximal contractions by CaCl_2_ (10 mM) were 1.93 ± 0.11 g, 1.67 ± 0.27 g, 0.99 ± 0.10 g, 0.35 ± 0.12 g, and −0.18 ± 0.05 g in the absence and presence of LEL (100, 200, 500, and 1000 μg/mL), respectively (Figure 4).

**Figure 5:**
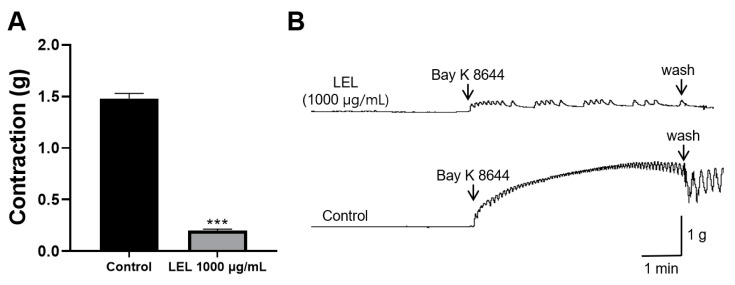
Effect of LEL on Ca^2+^ influx via L-type Ca^2+^ channel. LEL were applied to the rings (1000 µg/mL) before inducing contractions using Bay K8644 (10 µM). (**A**) Contraction response by Bay K8644 and (**B**) representative tracing. Data are presented as mean ± SEM (*n* = 5). *** *p* < 0.001 vs. control.

To investigate whether LEL has an inhibitory effect on L-type Ca^2+^ channels, the rings were constricted using Bay K8644 (an L-type Ca^2+^ channel agonist) in the rings incubated with or without LEL. Rings incubated with LEL (1000 μg/mL) showed a significant inhibitory effect on the contractions by Bay K 8644 (Figure 5).

### 3.5. Role of K^+^ Channels in LEL-Induced Vasorelaxant Activity

**Figure 6:**
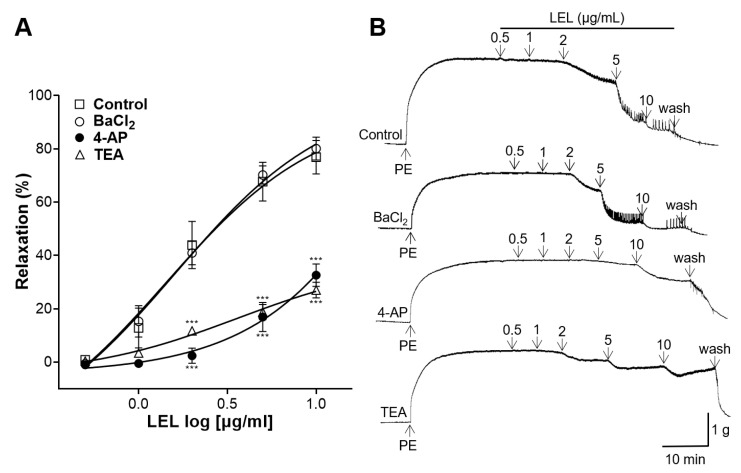
Effect of LEL on aortic rings with induced contraction by PE (1 μM) without (Control) or with the incubation of BaCl_2_ (10 μM), 4-AP (1 mM), or TEA (1 mM). (**A**) Relaxation response to LEL and (**B**) representative tracing. Data shown are the mean ± SEM (*n* =5–6). *** *p* < 0.001 vs. control.

One of the following were applied to the aortic rings: inward rectifier K^+^ channel blocker, BaCl_2_, voltage-dependent K^+^ channel blocker, 4-AP, or large-conductance Ca^2+^-activated K^+^ channel blocker, TEA. LEL resulted in the reduced relaxation of the aortic rings, which had undergone an induced contraction by PE, with the pre-incubation of 4-AP and TEA when compared to the control; however, the incubation with BaCl_2_ showed no significant effects on the LEL-induced vasorelaxant activity in aortic rings contracted with PE (Figure 6).

### 3.6. Effect of LEL on Rings Constricted by Angiotensin II

**Figure 7:**
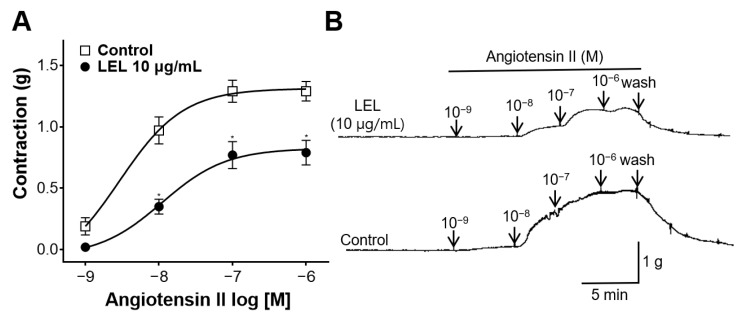
Effect of LEL on rings constricted by angiotensin II. (**A**) Contraction response to angiotensin II and (**B**) representative tracing. Data shown are the mean ± SEM (*n* = 4–5). * *p* < 0.05 vs. control.

We assessed whether LEL inhibited the constrictive action of angiotensin II in thoracic aortic rings. The cumulative application of angiotensin II induced concentration-dependent contractions, and LEL (10 μg/mL) significantly attenuated these contractions (Figure 7). The maximal contractions by angiotensin II (10^−6^ M) of the aortic rings incubated without or with LEL were 1.29 ± 0.08 g and 0.79 ± 0.10 g, respectively.

### 3.7. Hypotensive Effect of LEL

**Figure 8:**
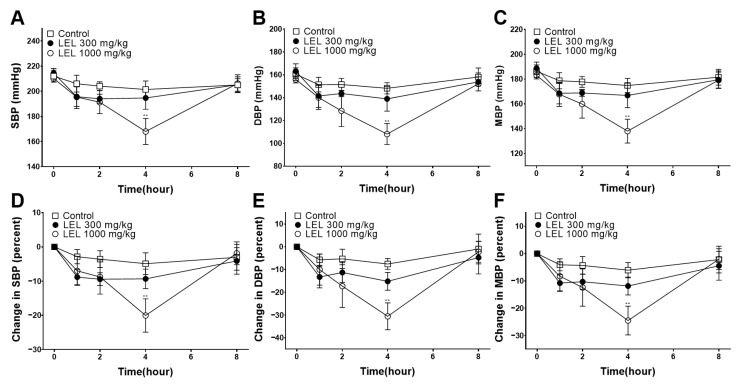
The effect of LEL on blood pressure. (**A**) SBP, (**B**) DBP, (**C**) MBP, (**D**) percent changes in SBP, (**E**) percent changes in DBP and (**F**) percent changes in MBP. Data shown are the mean ± SEM (*n* = 5). ** *p* < 0.01 vs. control.

The blood pressure after the oral administration of LEL to the SHRs are shown in Figure 8. 1000 mg/kg of LEL promoted a marked hypotensive effect in the SHRs compared to the control. The lowest was measured 4 h after the administration of 300 and 1000 mg/kg LEL, respectively. The SBP and DBP were reduced by −9.34 ± 2.86% and −15.19 ± 3.92%, respectively, in the LEL 300 mg/kg group at 4 h after administration. However, there was no significant difference with the control group. The SBP and DBP were reduced by −20.06 ± 4.87% and −30.58 ± 5.92%, respectively, in the LEL 1000 mg/kg group at 4 h after administration. The mean arterial blood pressure (MBP) also reduced significantly by −24.54 ± 5.28% in the LEL 1000 mg/kg group at 4 h.

## 4. Discussion

The effects of LEL on isolated aorta were investigated in this study. The cumulative administration of LEL promoted vasorelaxant activity of endothelium-intact and endothelium-absent rings in a dose-dependent manner. LEL induced a significant vasodilator effect with an E_max_ value of 73.07 ± 5.50% at 10 μg/mL in the endothelium-intact rings and 97.29 ± 0.39% at 1000 μg/mL in the endothelium-denuded rings. Our results indicate that the vascular relaxing effect of LEL is related to both the endothelium-dependent and endothelium-independent pathways. In addition, our results show that the vasodilatory effect of LEL acts primarily through an endothelium-dependent mechanism at relatively low doses, whereas at high concentrations, an endothelium-independent mechanism also acts.

After confirming the vasorelaxant effect of LEL, the endothelium-dependent mechanism in the vasorelaxant effects involved was investigated. The vascular endothelial cells are the primary regulators of vascular tone by various vasoactive agents, such as NO, prostacyclin (PGI_2_), or endothelin-1 [21]. The major endothelial-derived vasodilator substances are NO and PGI_2_ [21]. NO is a vasodilator that produces its effects mainly through the activation of sGC, which in turn promotes cGMP synthesis in VSMCs [22]. PGI_2_, produced by COX, induces vasodilation and activates adenylate cyclase, which generates cyclic adenosine monophosphate (cAMP) [23]. Increased cGMP or cAMP levels lead to the relaxation of VSMCs and subsequent vasodilation [24]. To reveal the role of NO and PGI_2_ in LEL-induced vasorelaxant activity, the aortic rings were incubated with L-NAME (an NO synthase inhibitor) or indomethacin (a COX inhibitor). Our results revealed that L-NAME treatment decreases the relaxation of the aortic rings; however, incubation with indomethacin did not display any significant effects on LEL-induced relaxation. To further elucidate the NO-related mechanisms related with the vasorelaxant effects of LEL, the rings were incubated with ODQ (an sGC inhibitor) or MB (a cGMP inhibitor). Our results showed that ODQ and MB pretreatment resulted in decreased relaxation compared to that of the control. Thus, our study shows that the mechanism underlying LEL-induced vasorelaxation involves the NO–sGC–cGMP pathway but is independent of PGI_2_.

In VSMCs, contractions are primarily controlled by the intracellular concentration of Ca^2+^, which activates and phosphorylates myosin light chain kinase, thereby enabling myosin to interact with actin [25]. Therefore, vasorelaxation in VSMCs can be achieved by reducing the intracellular Ca^2+^ concentration, inhibiting Ca^2+^ influx, promoting Ca^2+^ efflux, or increasing the uptake of Ca^2+^ into intracellular stores [26]. In the present study, we assessed the inhibitory effects of LEL on Ca^2+^ influx through Ca^2+^ channels. Aortic segments were pre-incubated with PE before treatment with CaCl_2_ (0.1, 0.3, 1, 3, or 10 mM). LEL (200, 500, and 1000 μg/mL) significantly decreased Ca^2+^-induced aortic ring constriction. PE-induced contraction involves various Ca^2+^ channels, such as L-type voltage-gated, receptor-operated, and store-operated Ca^2+^ channels [27]. Therefore, our study indicated that LEL can block the Ca^2+^ channels at high concentrations. LEL at concentrations of 100 µg/mL did not show a significant effect of blocking Ca^2+^ influx compared to the control group, and a significant inhibitory effect of LEL on Ca^2+^ influx was observed with doses ≥ 200 µg/mL (Figure 4). When the aortic rings were absent from the endothelium, significant vasorelaxation by LEL was observed with doses ≥ 200 μg/mL (Figure 1). Therefore, our study suggests that the Ca^2+^ channel-blocking effect of LEL is independent of the endothelium.

Among the various Ca^2+^ channels, L-type Ca^2+^ channels are crucial for initiating and maintaining VSMC contraction [28]. Additionally, owing to their wide distribution throughout the cardiovascular system, they are major pharmacological targets in treating hypertension and CVDs [29]. The inhibitory effects of LEL on L-type Ca^2+^ channels were investigated using Bay K8644, an L-type Ca^2+^ channel agonist. LEL (1000 μg/mL) exhibited a significant effect in inhibiting the contraction induced by Bay K8644. Therefore, our results indicate that LEL has an inhibitory effect on L-type Ca^2+^ channels.

The activation of K^+^ channels is a key mechanism in achieving blood vessel relaxation, facilitating increased blood flow and decreased vascular resistance [30]. Several K^+^ channels are related in this process, including inward rectifier K^+^ channels, voltage-dependent K^+^ channels, and large-conductance Ca^2+^-activated K^+^ channels [31]. The activation of these K^+^ channels allows for the efflux of K^+^ out of VSMCs and induces a hyperpolarization of the cell membrane and a reduction in the activity of Ca^2+^ channels, leading to reduced vasocontraction and thereby promoting relaxation [32]. Additional experiments were undertaken to elucidate the involvement of K^+^ channels in LEL’s vasorelaxant effects. Aortic rings were incubated with 4-AP (a voltage-dependent K^+^ channel blocker), TEA (a large-conductance Ca^2+^-activated K^+^ channel blocker), or BaCl_2_ (an inward rectifier K^+^ channel blocker). Our results showed that 4-AP and TEA treatment resulted in reduced relaxation of the aortic rings compared to the control; however, incubation with BaCl_2_ did not exert any significant effects on LEL-induced vasorelaxation. Therefore, our study suggests that the mechanism of LEL-induced vasorelaxation involves K^+^ channels, particularly voltage-dependent and large-conductance Ca^2+^-activated K^+^ channels.

The role of angiotensin II was investigated to further explore the vasorelaxant effects of LEL. Angiotensin II, the major peptide in the renin–angiotensin system, is a potent vasoconstrictor that increases resistance of blood vessels and blood pressure [33]. The acute stimulation of angiotensin II induces the immediate vasoconstriction by increasing the intracellular Ca^2+^ concentration [33]. In our experiments, LEL-treated aortic rings showed significantly attenuated angiotensin II-induced contractions, indicating that LEL inhibited the constrictive action of angiotensin II.

Finally, the effect of LEL on blood pressure in the SHRs was investigated. The SHRs represent an established genetic model of hypertension developed by selective breeding which naturally exhibit high blood pressure and are similar in the development of essential hypertension with humans [34]. SHRs are useful for evaluating the effectiveness of antihypertensive drugs or interventions because of their predictable hypertensive phenotypes and similar responses to antihypertensive agents [35]. In our study, the oral administration of 1000 mg/kg LEL showed a significant hypotensive effect in the SHRs compared to the control. The lowest reduction rates in SBP and DBP (−20.06 ± 4.87% and −30.58 ± 5.92%, respectively) were measured 4 h after administration in the LEL 1000 mg/kg group. Moreover, MBP showed the highest rate of decrease by −24.54 ± 5.28% in the LEL 1000 mg/kg group at 4 h. Our results provide preliminary evidence that LEL alleviates hypertension. Adverse effects, such as sudden death or weight loss >20%, were not observed following administration. Further research, including long-term administration and toxicity testing, is warranted to validate the use of LEL in hypertension treatment.

Plants and their natural compounds have shown potential in the treatment of hypertension through various mechanisms [36]. Our study demonstrated that LEL exhibits a vasorelaxant effect through the NO/cGMP pathway, blocking Ca^2+^ channels, activating K^+^ channels, and inhibits the constrictive action of angiotensin II. Blood pressure is regulated by the tone of the blood vessels, and relaxation of VSMCs leads to the dilation of the vessels and a reduction in the blood pressure [31]. This study demonstrated the vasodilatory properties and mechanisms of action of LEL in isolated rat thoracic aortas. The thoracic aorta of rats is the most commonly used model for studying vasodilation effects because of its accessibility and the ability to investigate mechanisms related to the endothelium and VSMCs [37]. However, considering that resistant arteries are key elements of vascular resistance and blood pressure, additional experiments are required to elucidate the vasorelaxant effects of LEL on smaller arteries [38]. In addition, LEL resulted in the reduction in blood pressure in the SHRs, which are an established model of essential hypertension. Overall, LEL may be a valuable source for the treatment of hypertensive diseases. In particular, plant leaves are easily accessible and have a history of usage in traditional medicine; therefore, LEL could be an effective and affordable alternative for treating hypertension [39]. Three marker compounds, methyl lucidone, methyl linderone, and kanakugiol, have been identified as the major contributors to the therapeutic effects of LE [40]. However, this study did not analyze the components of LEL. Therefore, further research is required to identify the active components of LEL for an enhanced understanding and potential commercial utilization.

## 5. Conclusions

This study demonstrated the anti-hypertensive properties of LEL. LEL exhibits a vasorelaxant effect through both endothelium-dependent and endothelium-independent mechanisms. The related mechanisms include the activation of NO–sGC–cGMP pathway, blocking the Ca^2+^ influx, opening K^+^ channels, or inhibiting the angiotensin II-induced contraction. In addition, LEL oral administration resulted in the significant reduction in blood pressure of SHRs. Therefore, our study suggest that LEL may be an effective alternative for the treatment of hypertensive diseases. However, further researches on the active components and toxicity of LEL are needed.

## Data Availability

The original contributions presented in the study are included in the article; further inquiries can be directed to the corresponding author.
